# Biocomposites from poly(3-hydroxybutyrate-co-3-hydroxyvalerate) and lignocellulosic fillers: Processes stored in data warehouse structured by an ontology

**DOI:** 10.1016/j.dib.2022.108191

**Published:** 2022-04-22

**Authors:** Mélanie Munch, Patrice Buche, Stéphane Dervaux, Amélie Breysse, Marie-Alix Berthet, Grégoire David, Sarah Lammi, Fleur Rol, Amandine Viretto, Hélène Angellier-Coussy

**Affiliations:** a IATE, INRAE, Montpellier SupAgro, Université de Montpellier, Montpellier F-34060, France; b IATE and I2M, INRAE, Université de Bordeaux, Talence 33405, France; c LIRMM, CNRS, INRIA GraphIK, Université de Montpellier, Montpellier F-34060, France; d UMR MIA-Paris, AgroParisTech, INRAE, Université Paris-Saclay, Paris F-75005, France

**Keywords:** Biocomposites, Tensile properties, Lignocellulosic fillers, Food packaging, Water vapour permeability

## Abstract

Due to the rising amount of plastic waste generated each year, multiple questions are emerging about their harmful long-term effects on the environment, the eco-systems and human health. One possible strategy to mitigate these issues is to substitute conventional plastics by materials fully biodegradable in natural conditions, such as poly(3-hydroxybutyrate-co-3-hydroxyvalerate) (PHBV). In order to decrease the overall cost and environmental impact of PHBV-based materials while modulating their technical performance, PHBV can be combined with lignocellulosic fillers. In this article, a total of 88 formulations of PHBV-based biocomposites has been collected, distributed over 5 interdisciplinary projects involving computer scientists, data scientists and biomass processing experts for food and bio-based material production. Available data concern the technical process descriptions, including the description of each step and the different observations measured.

These data are stored in a knowledge base that can be queried on the Web.

## Specifications Table

**Table:** 

Subject	Polymers and plastics
Specific subject area	Lignocellulose-based biocomposites for food packaging
Type of data	Table
How the data were acquired	Characterization of lignocellulosic fillers. Lignocellulosic particles characterized and used as fillers for the production of biocomposites were either purchased as commercial grades, *i.e.* cellulose [10] and wood fibres [9,10] or produced by dry fractionation of raw biomasses, *i.e.* wheat straw [4,5], vine shoots [8], olive pomace [5,6], and green park and garden waste [9]. Lignocellulosic fillers were characterized in terms of biochemical composition (cellulose, lignin, hemicellulose and ashes contents) using biochemical analyses, and morphology (apparent median diameter and span value) measured by laser granulometry. Characterization of biocomposites. Materials were produced following two processing steps: first, a compounding step to mix lignocellulosic particles with the PHBV polymer matrix; and then, a shaping step to get either thermopressed films [4,^5^,^7^,10] or injection moulded samples [8,9]. Biocomposites were characterized in terms of thermal properties (melting and crystallization temperatures) assessed by differential scanning calorimetry (DSC) analysis, thermal stability (temperatures of thermal degradation) assessed by thermogravimetric analysis (TGA), mechanical properties (Young's modulus, strain at break and stress at break) assessed by tensile tests and water vapour permeability.
Data format	Analyzed
Description of data collection	Data are structured by the Process and Observations Ontology (PO²), which allows describing transformation processes in a knowledge base available on the Web 88 biocomposites produced from the extrusion of 31 lignocellulosic fillers and 7 polymers (PHBV) are presented in 5 tables: Table 1: Description of the lignocellulosic fillers Table 2: Description of the PHBV Table 3: Description of the produced biocomposites Table 4: Itineraries of production of the lignocellulosic, from the raw material to the powder intended to be extruded with PHBV Table 5: Itineraries of production of the biocomposites packaging, from the extrusion to the thermoforming process
Data source location	INRAE, PLANET-IATE, 1208. Agropolymers and Emerging Technologies Facility, Montpellier FR-34060, France
Data accessibility	Repository name: INRAE dataverse (https://data.inrae.fr/) Data identification number: 10.15454/G3GBJC Direct URL to data: https://data.inrae.fr/dataset.xhtml?persistentId=doi:10.15454/G3GBJC

## Value of the Data

• This dataset provides an overview of the impact of lignocellulosic filler size, composition and content on the final performance of PHBV-based biocomposites based on the characterization of a total of 88 formulations stemming from different research projects.
• Such compiled dataset allows to highlight which filler parameters are impacting the most the final performance of biocomposites, with greater robustness than in each isolated study.
• These data can be used to produce tailored-made biocomposites based on a reverse engineering approach.
• All the stakeholders of the biocomposites field can benefit from this data, from the manufacturers to the end-users.
• This dataset is described by a reusable domain ontology enriched with specific vocabulary from the biocomposites domain.

## 1 Data Description

Data are stored in two datasets (see Data accessibility in the Specification Table above): the first one contains the biocomposites food packaging description, and the second the domain ontology with a specific vocabulary used to structure the first*.* This dataset uses a vocabulary defined with experts and described by PO² (Process and Observation Ontology) [1], an ontology dedicated at its core to the representation of transformation processes through the definition of steps, relations between those and their associated observations. In our case, specific domain vocabulary has been elicited and used to define precisely the different technologies used for the biomass treatment and the biocomposites production. This vocabulary can be reused for others projects on the domain [2,3].

The first dataset presents five tables to provide a general overview of the itineraries used to produce biocomposites, and the associated scripts and/or queries to create them. All queries are expressed in the SPARQL language, dedicated to interrogate data structured by ontologies. Queries can either be directly executed using the accessible SPARQL endpoint (http://quantum.agroparistech.fr/graphdb/repositories/Composite_making_process), or formulated and executed through our web-service SPO²Q (http://quantum.agroparistech.fr/spoq) which also provides an interactive form to build queries without relying on SPARQL syntax. In a general manner, all queries introduced in this paper are presented in two ways: directly as a SPARQL query in a file, and as query executable from SPO²Q.

In Table 1, we present the 31 lignocellulosic fillers used through the four projects. For each, we indicate the process in which it was defined, as well as its ID used in the biocomposites’ denomination. We then detail their main biochemical composition (cellulose, hemicellulose, lignin and ashes contents), as well as some morphological characteristics assessed by laser granulometry. The results presented in this table have been queried using Query 1. Biomass description. To be noted, some units have been changed for a better readability of the file.

In Table 2, we characterize the 7 batches of poly(3-hydroxybutyrate-co-3-hydroxyvalerate) (PHBV) used through the different projects. For each, we present certain measures important for the biocomposites packaging's characteristics’ evaluation: tensile properties (such as the strain at break, the stress at break and the Young's modulus); thermal properties (crystallization and melting temperatures); thermal degradation temperatures (onset degradation temperature and temperature at the maximal rate of degradation); and water vapour permeability. To be noted, the PHBV's denominations (PHBV or PHBV2) are intern to the projects and have only been defined for distinguishing them when multiple batches were used in a same process. Similarly to Table 1, the results produced in Table 2 have been obtained through Query 2, with some units’ conversion when necessary.

In Table 3, we describe the 88 biocomposites produced using the biomasses and polymers presented in Tables 1 and 2. For a given process, a composite is labelled using the biomass and PHBV's ID, as well as the weight filler content (in brackets). All biocomposites are described by the same characteristics as the PHBV matrix. Results were produced by Query 3.

Table 4 presents the succession of processing steps (grinding and/or drying) for each itinerary leading to the production of a lignocellulosic filler, starting from the raw biomass and ending with the powder ready to be extruded with the polymer. Given a biomass, steps are numbered to indicate the chronology; when two steps happen in parallel, they are distinguished by a second number. For instance, the UPRF sample of the Resurbis project is prepared from different sub-samples produced in steps 1., 1.2, 1.3, … which are mixed in step 2. For each step, the available observations’ measures are indicated. Due to the complexity of the data presented in this table, we need to compile the outputs of a SPARQL query to organize the data in a readable and easy-to-use way. This script as well as the dedicated SPARQL query are presented in Script 1. It is written in Python, and give an example on how to integrate the SPARQL endpoint in a code for extracting and manipulating the data. To be noted, the USABLE project uses commercial lignocellulosic fillers of pure cellulose, which were already prepared, and thus are not represented in this table.

Finally, Table 5 presents the itineraries for producing the biocomposites materials, starting from the extrusion step (mixing the components using a thermo-mechanical process to obtain compounds) and ending with the shaping step. A numeration similar to the one used in Table 4 is used. This table has also been produced by a Python script presented in Script 2.

## 2 Experimental Design, Materials and Methods

### 2.1 Characterization of lignocellulosic fillers

The weight fractions of the main biochemical constituents of lignocellulosic fillers, *i.e.* cellulose, lignin and hemicellulose, were determined by biochemical analysis following the protocol described in Viretto et al. [9]. The ashes content was calculated from thermogravimetric analysis [9]. Filler apparent median apparent diameter and span values were determined from volume size particle distributions obtained by laser granulometry, as described in Berthet et al. [4].

### 2.2 Characterization of biocomposites

Crystallization and melting temperatures of biocomposites were, respectively, determined from the cooling ramp and the second heating ramp of thermograms recorded by DSC [8]. Thermal degradation temperatures were determined from TGA thermograms. Both the onset and the peak temperatures were measured, which correspond, respectively, to the beginning of the degradation phenomenon and to the moment at which the degradation rate is maximum [8]. Mechanical properties, *i.e.* nominal elongation at break, strength at break, and Young's modulus, were determined at room temperature from stress-strain curves obtained by uniaxial tensile tests. Different testing conditions (type of texture analyzer, cross-head speed, relative humidity) were considered depending on the research project: MALICE / ECOBIOCAP [3], [4], [5], [6], NoAW [7], RESURBIS [8], and USABLE [9]. Finally, water vapour permeability was measured in the case of films according to the protocol detailed by Berthet et al. [4].

## Ethics Statements

This work neither involves human subject nor animal experiments.

## CRediT authorship contribution statement

**Mélanie Munch:** Data curation, Writing – original draft, Writing – review & editing. **Patrice Buche:** Methodology, Software, Data curation, Writing – review & editing. **Stéphane Dervaux:** Software, Data curation. **Amélie Breysse:** Investigation. **Marie-Alix Berthet:** Investigation. **Grégoire David:** Investigation. **Sarah Lammi:** Investigation. **Fleur Rol:** Investigation. **Amandine Viretto:** Investigation. **Hélène Angellier-Coussy:** Conceptualization, Methodology, Investigation, Writing – original draft, Writing – review & editing.

## Declaration of Competing Interest

The authors declare that they have no known competing financial interests or personal relationships that could have appeared to influence the work reported in this paper.

## Data Availability

Itinerary Description for biocomposites from poly(3-hydroxybutyrate-co-3-hydroxyvalerate) and lignocellulosic fillers (Original data) (Dataverse).
